# A systematic review of heterosexual anal intercourse and its role in the transmission of HIV and other sexually transmitted infections in Papua New Guinea

**DOI:** 10.1186/1471-2458-13-1108

**Published:** 2013-12-01

**Authors:** Angela Kelly-Hanku, Andrew Vallely, Wing Young Nicola Man, David Wilson, Greg Law, Richard Gray

**Affiliations:** 1 Sexual and Reproductive Health Unit, Papua New Guinea Institute of Medical Research, Goroka, Papua New Guinea; 2 International HIV Research Group, School of Public Health and Community Medicine, University of New South Wales, Sydney, Australia; 3 Kirby Institute, University of New South Wales, Sydney, New South Wales, Australia; 4 Faculty of Health Sciences, University of Sydney, Sydney, New South Wales, Australia; 5 STI Clinical Specialist and Advisor, Port Moresby, Papua New Guinea

**Keywords:** Condoms, Heterosexual anal intercourse, Human immunodeficiency virus, Papua New Guinea, Sexually transmitted infections

## Abstract

**Background:**

Papua New Guinea (PNG) has a high burden of sexually transmitted infections (STIs) and the highest adult HIV prevalence in the Pacific region. Despite this burden of disease, heterosexual anal intercourse (HAI) has rarely been considered. Given the increasing number of, and interest in, behavioural surveys in PNG and the changing nature of PNG’s HIV epidemic, it is timely to conduct a systematic review of HAI in PNG order to improve sexual health.

**Methods:**

We performed a systematic review of HAI in PNG as reported in peer-reviewed and non-peer-reviewed publications for the period 1950–May 2012. The search strategy identified 475 publications. After screening by geographical location, topic and methodology, we identified 23 publications for full text review, following which 13 publications were included in the final review. Using data from the review, we performed a risk equation analysis to demonstrate the potential impact of HAI on HIV acquisition and incidence in PNG.

**Results:**

There is a paucity of well-informed behavioural research on HAI in PNG. Inconsistency in key questions on HAI made it impossible to conduct a meta-analysis. The data available on HAI shows that it is practiced in all geographical areas and among all populations. Of those who reported HAI, rates varied from as low as 8% to as high as 77% depending on the recall period and partner type. Condom use during HAI was consistently low. Our risk equation analysis indicates that even if only 20% of females engage in HAI, and only 10% of sex acts involve HAI, the total number of new HIV infections among females would be 40% greater than if vaginal intercourse only occurred.

**Conclusions:**

Our findings of indicate that HAI may be an important driver of the HIV epidemic in PNG. In order to improve the sexual health of Papua New Guineans, efforts are required to improve behavioural surveillance of HAI as well as develop national HIV/STI programing and policy to better address the risks associated with unprotected HAI.

## Background

Irrespective of whether penile-anal intercourse is occurring between a male and a female or two males, penile-anal intercourse is associated with greater risk of HIV transmission than penile-vaginal intercourse [1-7]. The risk of transmission during unprotected anal intercourse has been estimated to be 10 times greater than during unprotected vaginal intercourse [8-10]. Some estimates suggest the risk could be as high as 20-fold [11]. While some caution is required for pooled transmission probabilities, it is clear that unprotected HAI poses greater risk for females than does vaginal intercourse [7]. In heterosexually driven epidemics, HIV transmission is often presumed synonymous with vaginal intercourse, as there is no distinction between risk behaviours [12]. Despite the higher transmission risk of HIV from males to females during anal intercourse, behavioural surveys rarely acknowledge HAI.

### Heterosexual anal intercourse: a global perspective

Despite the risks associated with unprotected HAI, HAI in general and unprotected HAI specifically have received limited attention [5,12-14]. One study of American men and women found that 35% of those surveyed had engaged in HAI [15] while another among youth aged 15-21 years in three US cities found 16% had engaged in HAI in the previous three months [16]. Of women attending a US clinic, Bolling et. *al*. reported that 72% of women had ever engaged in HAI, with 23% reporting regular HAI [14]. Another study reported that 20% of American women were at high risk of HIV, reporting having had HAI with a variety of partners, including paying clients [17]. A study among Puerto Rican college students in the US found that 44% of males and 32% of females practiced anal sex [5]. In the context of a randomised study including young black and Latina women in the US, it was found that 23% and 35% respectively reported having ever engaged in HAI, with 47% and 61% doing so in the preceding two months respectively [18].

Certain sub-populations appear to engage in HAI more frequently than others, including bisexual women [19] and men [20,21], women whose sexual partner injects drugs [19], women selling or exchanging sex [19,22-28] and some ethnic groups [5]. For example, a study among female sex workers (FSW) in South Africa found that 42.8% reported HAI with their clients [24], but lower rates have been reported in other African countries such as Rwanda (5.5%) and Kenya (4.3%) [29].

Despite the increased HIV acquisition risk associated with HAI, in many settings HAI attracts stigma and HIV and STI prevention campaigns largely ignore this issue [12]. Subsequently, people’s knowledge and awareness about anal sex and HIV transmission is lacking [12]. This is important because HAI may play a larger role in HIV transmission in the general population than previously thought [7]. Reported condom use for vaginal intercourse is higher than for HAI in several studies [5,30]. For example, a study among women in New York reported that with casual male partners 96% never used condoms during HAI compared to 33% during vaginal intercourse [31]. It is estimated that in the USA the number of women practising unprotected anal intercourse is 7 times higher than the number of men having unprotected anal intercourse with men [5]. Similarly, a study of Australian bisexual men showed a relatively low rate of condom use for HAI with only 29% of men having reported using a condom every time they had HAI [21].

To date the research on HAI is dominated by behavioural studies and quantitative measures. That said, increasing attention is being afforded to the cultural context and meanings of HAI [12,24,32-36].

### HIV and heterosexual anal intercourse: Papua New Guinea

Until recently, available data indicated that PNG might have a generalised HIV epidemic. However, more recent and improved national surveillance data suggest that the epidemic has progressed less rapidly than feared, with ~1% of the adult population infected [37]. PNG is now classified as having a concentrated, or at least a mixed, HIV epidemic. Rates of infection vary between populations and across the country with the greatest burden in the Highlands region, Morobe Province and the National Capital District, where over 90% of new diagnoses come from seven of the 22 provinces [37]. Certain ‘key affected populations’ at increased risk of HIV infection have been identified including men who have sex with men and people involved in the selling and exchange of sex [38]. For example, in a respondent driven sampling (RDS) study of sex workers in PNG’s capital Port Moresby, 16.9% were identified as HIV-positive through rapid HIV testing [39].

Cultural notions of pollution, kinship, gender and Christianity have historically prescribed sexual behaviour in PNG [40-43]. As is true of all sexual practices, anal sex is deeply imbued with meaning [12]. Anthropologists working in PNG have long described traditional initiation rituals of manhood that involved sex between males, including both oral and anal sex [43-48]. Discussion of anal intercourse in PNG has thus almost exclusively referred to male-to-male sex, usually in the context of the ‘ritualised homosexual’ [45,46].

While literature on HAI in many developing/transitional countries is increasing, there is limited literature on HAI in PNG. Importantly, except in the context of male-to-male sex, there is almost no discussion of HAI in HIV prevention work in PNG, the prevention of STIs from HAI, or the treatment of anorectal STIs. Furthermore, at present there is no evidence to estimate the role the unprotected HAI plays in HIV acquisition amongst PNG women nor is there any evidence to inform improved and specialist public health policy and programming to address HAI including clinical care and social and behavioural research.

In this paper, we present results from the first systematic review of HAI in PNG, and the findings of a risk equation analysis to estimate the contribution of HAI to female HIV acquisition in this setting. The paper concludes by discussing the implications of these findings for public health policy and programming in PNG.

## Methods

### Literature searches

A systematic review of the literature was conducted according to PRISMA guidelines [49]. We searched PubMed (1950-May 2012), JTSOR (1950-May 2012) and Web of Knowledge (1950-May 2012) to identify peer-reviewed studies using the Medical Subject Headings (MeSH) terms: ‘Papua New Guinea’; ‘PNG’; ‘sexual behavior’; ‘sexual behaviour’ and; ‘sexual practice’. No language restrictions or other limitations were placed in the searches. Sexual behaviour data from published behavioural surveillance and other research reports were obtained on the Internet (http://www.sphcm.med.unsw.edu.au/centres-units/international-hiv-research-group/publicationshttp://www.nri.org.pg/publications/recent_publications.htm). Those that were not available electronically on-line they were obtained from the PNG National AIDS Council Secretariat Research Coordination Unit Resource Library, which has electronic and hard copies of all HIV-related research reports in the country. All studies had obtained the appropriate ethical approval with all data anonymous and consent obtained. The lead author completed this literature search.

### Selection of studies

We excluded studies that did not include quantitative data on sexual behaviour. All studies had to report behavioural data on the sexual practices of Papua New Guineans, either amongst the general population or key affected populations such as sex workers.

#### Data extraction and data analysis

Data on study design, sex, study population, age of sample, and geographical location of study were extracted. Further extractions included types and frequency of sexual behaviour, condom use for specific sexual practices, recall periods and experiences of and knowledge of STIs. The lead author completed data extraction. Due to the paucity of the data a meta-analysis was not possible and therefore confidence intervals were not calculated. Because the survey questions on HAI were not standardised we cannot present the data in a forest plot.

### Impact of HAI: risk equation analysis

We performed a simple risk equation analysis to investigate the potential impact of HAI on PNG’s HIV epidemic and highlight the importance of measuring the behaviour associated with HAI. We derived the risk equation using a binomial equation and standard HIV modeling techniques [50,51]. The equation calculates the cumulative probability over all sexual acts of an individual acquiring HIV each year. It assumes a homogenous population with each person having the same average characteristics and behaviours. Such an analysis also ignores the additional transmission from people who acquire HIV during a given year, and has a number of other limitations, but provides an estimate of the annual risk of infection. This approach is used extensively for understanding short-term HIV incidence [52]. For HIV-negative females the cumulative probability of acquiring HIV over multiple unprotected sexual acts of vaginal or anal intercourse is:

(1) $$\beta =1-\overset{\text{anal}\;\text{acts}}{\overbrace{{\left(1-\beta_{a}\right)}^{n_{a}}}}\overset{\mathrm{vaginal}\;\mathrm{acts}}{\overbrace{{\left(1-\beta_{v}\right)}^{n_{v}}}}$$

where $n_{a}=np_{a}\left(1-p_{c}^{a}\right)P_{\mathrm{HIV}}$ and $n\left(1-p_{a}\right)\left(1-p_{c}^{v}\right)$ are the number of unprotected anal and vaginal acts, respectively, *β*_*v*_ is the male-to-female transmission probability during unprotected vaginal intercourse; *β*_*a*_ is the male-to-female transmission probability during unprotected anal intercourse. In our equations, *n* is the total number of sexual acts, *p*_*a*_ is the proportion of sexual acts that involve anal intercourse, $p_{c}^{v}$ is the proportion of vaginal intercourse acts where a condom is used, $p_{c}^{a}$ is the proportion of anal intercourse acts where a condom is used, and *P*_*HIV*_ is the prevalence of HIV in male partners. For calculation purposes, we assume condoms are 100% protective.

By entering appropriate values, we calculated the risk of HIV infection under various conditions and for specific groups of females. We estimated the annual incidence by multiplying the annual risk by the population size. The impact of HAI on HIV incidence in the general population was estimated by dividing the female population into those who do and do not engage in HAI and calculating the incidence in each population. Our calculations used the parameter values in Table 1. These values are based on available behavioural and epidemiological data for PNG (referenced in the footnotes in Table 1)—we estimated the percentage of women who engage in HAI and their condom use during intercourse using the quantitative results obtained in this review (Table 2 and Table 3). Given the paucity of data for PNG, most of the parameter values are assumptions. However, they broadly reflect the behavioural and epidemiological characteristics of the PNG population and allow us to assess the contribution of HAI to the HIV epidemic [refs]. The last author led these calculations.

**Table 1:** Parameter values used in calculations

**Model parameter**	**Representative value**
Per-act male-to-female HIV transmission probability during vaginal intercourse^a^	0.0008
Per-act male-to-female HIV transmission probability during anal intercourse^a^	0.0143
**Anal sex behavior based on review results**	
Probability of condom use during vaginal intercourse–Women in general population^b^	20%
Probability of condom use during anal intercourse–Women in general population^b^	10%
Probability of condom use during vaginal intercourse–Women at high risk^b^	40%
Probability of condom use during anal intercourse–Women at high risk^b^	20%
Proportion of women in general population who engage in HAI each year^c^	20%
Proportion of women at high risk who engage in HAI each year^c^	50%
Proportion of sex acts that involve anal intercourse–Women in general population who engage in HAI^d^	10%
Proportion of sex acts that involve anal intercourse–Women at high risk who engage in HAI^d^	20%
**Behavioral and epidemiological characteristics for PNG**	
Number of sex acts per year–unmarried women in general population^e^	10
Number of sex acts per year–Women in general population with a regular partner	100
Number of sex acts per year–Women at high risk^e^	250
HIV prevalence in male partners^f^	1%

a: Male to female vaginal transmission based on [11]. For HIV transmission through HAI this is assumed to be the same as for male-to-male transmission. These values are from a systematic review for homosexual men in Australia and represent the probability of HIV transmission for receptive anal intercourse with ejaculation [53].

b: Condom use for HAI assumed based on data from our systematic review on condom use during last act (Table 1). From our review condom use for HAI at last HAI act varied between 4% and 19%, depending on population, but the number of people surveyed were small. Vaginal intercourse condom use assumed to have higher values based on our findings that it is universally higher than for HAI [5,30]. Women at high risk assumed to have a higher condom use than women in general population with values based on behavioral data from PNG reviewed in [54]. Based on this information we assumed condom use during last act of HAI to be 10% for the general population and 20% for women at high risk.

c: Assumed values obtained in our systematic review (Table 3). According to our review 18% to 24% of female youth out of school engaged in HAI in the last 12 months. For calculation purposes, we assume this is a reasonable representation of the behavior of women in the general population and assume 20% of females in the general population engage in HAI each year. Data for HAI in the last month, 3 months, and 6 months in Table 3 suggest HIA is substantially more common amongst FSW, generally over 50% for each period. We therefore assumed 50% of women at high-risk engage in HAI each year.

d: Assumption as there is no available frequency data for HAI. We assume HAI is more frequent for women at high risk.

e: Values based on behavioral data from PNG on the number of casual partners reviewed in [54].

f: Based on estimates from the National Department of Health [55].

Each parameter is given a representative value based on the data obtained from the systematic review or assumed where no data was found.

**Table 2:** Proportion of females that have engaged in HAI by recall period and study population

**Recall period**	**Population**	**Rate of HAI**	**Studies**
HAI in lifetime	Female sex workers	63% (n not provided)	[56]
		30% (HAI and oral sex)	[57]
		47.8% (n not provided)	[58]
	General Adult population	9.85% (n = 866)	[58]
	Female	8.4% (n = 428)	
	Male	11.3% (n = 438)	
	General Youth Population	9.6% (n not provided)	[58]
	Female	12.3% (n = 65)	
	Male	7.7% (n = 91)	
	Male adults	12% (n = 485)	[58]
	Male youth	11.7% (n = 384)	[58]
	Antenatal women	11.4% (n not provided)	[59]
	Female rural enclave workers	65 women (% not provided)	[60]
	Women	14.1% (n = 58)	[61]
HAI in last 12 months	Male truck drivers	31.4% (n not provided)	[62]
	Military men	59% (n not provided)	[62]
	Ramu Sugar workers	1% (n not provided)	[62]
	Lae Port Workers	13.5% (n not provided)	[62]
	Male out of school youth (married)	44.7% (n not provided)	[62]
	Male out of school youth (unmarried)	45.5% (n not provided)	[62]
	Female out of school youth (married)	23.4% (n not provided)	[62]
	Female out of school youth (unmarried)	18.5% (n not provided)	[62]
	STI Clinic patients	43.5% (n = 30)	[63]
	Male	37.9% (n = 11)	
	Female	47.5% (n = 19)	
HAI in last 6 months	People living with HIV	8.8% (n = 11)	[64]
	Sex workers with clients	46% (n = 273)	[39]
	Women with clients	46% (n = 201)	
	Men with clients	57% (n = 55)	
	Transgender with clients	30% (n = 17)	
	Sex workers with regular non-paying partners	58% (n = 240)	[39]
	Women with regular non-paying partners	56% (n = 167)	
	Men with regular non-paying partners	77% (n = 58)	
	Transgender with regular non-paying partners	33% (n = 15)	
	Sex workers with casual non-paying partners	53% (n = 201)	[39]
	Women with casual non-paying partners	48% (n = 131)	
	Men with casual non-paying partners	76% (n = 48)	
	Transgender with casual non-paying partners	52% (n = 22)	
HAI in last 3 months	Female sex workers	40% (n not provided)	[65]
HAI in last month	Female sex workers	57% (n not provided)	[65]
	FSW with one-time client	51% (n not provided)	
	FSW with regular client	53% (n not provided)	
	Who had paid for sex with a man	20% (n not provided)	
	Men who have sex with men	54% (n not provided)	[65]

**Table 3:** Condom use during HAI by population

**Condom use**	**Population**	**Rates of condom use**	**Studies**
Condom use frequency for HAI last month	Female sex workers	7% (frequency not stated)	[65]
Condom use frequency for HAI in last 6 months	Sex workers with clients (N = 272)	Every time 30%	[39]
		Almost every time 7%	
		Sometimes 56%	
		Never 6%	
	Women with clients (N = 200)	Every time 34%	
		Almost every time 7%	
		Sometimes 57%	
		Never 6%	
	Men with clients (N = 55)	Every time 30%	
		Almost every time 7%	
		Sometimes 57%	
		Never 3%	
	Transgender with clients (N = 17)	Every time 24%	
		Almost every time 6%	
		Sometimes 59%	
		Never 12%	
	Sex workers with regular non-paying partners (N = 239)	Every time 24%	[39]
		Almost every time 3%	
		Sometimes 59%	
		Never 14%	
	Women with regular non-paying partners (N = 166)	Every time 22%	
		Almost every time 2%	
		Sometimes 62%	
		Never 14%	
	Men with regular non-paying partners (N = 58)	Every time 29%	
		Almost every time 7%	
		Sometimes 48%	
		Never 16%	
	Transgender with regular non-paying partners (N = 15)	Every time 24%	
		Almost every time 3%	
		Sometimes 59%	
		Never 14%	
	Sex workers with casual non-paying partners (N = 200)	Every time 31%	[39]
		Almost every time 7%	
		Sometimes 58%	
		Never 5%	
	Women with casual non-paying partners (N = 131)	Every time 31%	
		Almost every time 6%	
		Sometimes 62%	
		Never 2%	
	Men with casual non-paying partners (N = 47)	Every time 36%	
		Almost every time 11%	
		Sometimes 40%	
		Never 13%	
	Transgender with casual non-paying partners (N = 22)	Every time 18%	
		Almost every time 0%	
		Sometimes 73%	
		Never 9%	
Condom use for HAI at last HAI act	STI Clinic Patients	11.6% (n = 8)	[63]
	Female	4.3% (n = 3)	
	Male	7.2% (n = 5)	
	Female rural enclave workers – Oil Search	18.2%	[66]
	Female rural enclave workers – WR Carpenters	4.5% (n = 3)	[60]
	Antenatal women	17.1%	[59]
	People living with HIV on ART (N = 11)	18.2%	[64]

The results reported are dependent on the survey question: Did not ask about condom use for reported HAI [56,57]; Asked about condom use frequency for HAI [39,65]; Asked about condom use frequency for HAI by partner type [39]; Asked about condom use for HAI at last HAI [59,60,63,64,66].

## Results

### Heterosexual anal intercourse in PNG: systematic review

Following the methods outlined in the PRISMA Guidelines, after the initial search was completed and the removal of duplicates was finalised, our search strategy resulted in 475 publications. After screening publications by geographical location, topic and methodology we excluded 452 publications leaving us with 23 for full review. Following a review of the full text, only 13 publications were included in the synthesis. As a result of the paucity of data, inconsistent recall periods and reporting of condom use we were unable to conduct a meta-analysis (See Additional file 1 for PRISMA flow chart).

Studies report HAI in most pre-specified population groups in PNG including people living with HIV. They document HAI in all four administrative regions of the country. Within the regions, the number of studies reporting HAI varies by province as an artefact of the number of sexual behaviour studies that included questions on HAI. For example, four separate studies report HAI in Morobe Province while only one reports HAI in East Sepik. Rates of HAI varied within populations within and across populations (Table 2) and therefore provinces. For example, while female youths in Oro Province reported no HAI, adult men and women and male youths in the province did [58]. HAI was not always disaggregated by province [64]. One study of FSW reported on HAI and penile-oral sex simultaneously making it impossible to discern what proportion of FSW engaged in HAI [57].

Condom use data for HAI is inconsistent. In some studies no rates of condom use for HAI are reported [58]. Where surveys report condom use, they indicate very low rates of condom use for HAI (Table 3) (we therefore assume similar results below 50% of high-risk women engage in HAI each year (Table 1) in our risk equation analysis)

Two studies provide the greatest detail on HAI in PNG [39,58]. *Askim na Save*, an integrated bio-behavioural study of sex workers (male and female) in Port Moresby, reported consistently high rates of HAI in the previous six months across all sex partner types [39]. Forty six percent of all sex workers reported HAI with clients, 53% reported HAI with casual non-paying partners and 58% reported HAI with regular non-paying partners (based on these and similar results below we assume 50% of high-risk women engage in HAI each year (Table 1) in our risk equation analysis). Importantly, of male sex workers both those who identified as a man and those who identified as transgender (born male but identify as transgender but without corrective surgery or hormone treatment) reported anal intercourse with females.

Condom use for HAI amongst the sex worker population was low. While 30% and 31% of sex workers in the study reported using a condom every time that had HAI with a client and casual non-paying partner in the last six months respectively, only 24% reported using a condom every time during HAI with a regular non-paying partner. Importantly, condom use in the last six months with a client and casual non-paying partner was lower for HAI than for vaginal intercourse. The report identifies some gender disparities in condom use. For example of women who sold sex, a greater proportion (34%) reported always using a condom for HAI with a client compared to male sex workers who identified as ‘transgender’ (24%) or as a ‘man’ (20%). Male sex workers (both ‘men’ and ‘transgender’) who sold sex reported lower rates of condom use for HAI than they did for anal intercourse with other males. Of male sex workers who identified as transgender, 42% reported that they used a condom every time they had HAI while 51% reported that they used a condom every time they had male-to-male anal sex. Of male sex workers who identified as a man, 20% reported that they used a condom every time they had HAI while 24% reported that they used a condom every time they had male-to-male anal sex [39]. Using this limited data, we assume condom use for anal intercourse is lower than for vaginal intercourse in our risk equation analysis (Table 1). We also assume females in the general population use a condom 10% of the time when engaging in HAI while females at high risk of HIV have a condom use of 20% during HAI (Table 1).

The second study, a baseline assessment for the PNG-Australia Sexual Health Improvement Program (PASHIP) shows that HAI is practiced by general adult populations, as well as youths and FSW [58]. The highest rates of HAI occur amongst FSW where 47.8% reported having ever had HAI. Following FSWs, both general population adults and youth from Southern Highlands Province reported the highest rates of lifetime HAI: varying between 18.1% and 30.5%. The remainder of the sample reported less than 15% with some reporting no HAI (female youths from Oro).

According to the PASHIP study there was no clear relationship between HAI and age in the general population but sex may be a factor [58]. For general population males, youth participants from Southern Highlands Province and East New Britain Province reported higher lifetime rates of anal intercourse compared to adult males (30.5% vs. 18.1% and 12.2% vs. 6.8% respectively). In Simbu Province, male adults reported over double the lifetime rate of anal intercourse than did male youth from the same province (13.9% vs. 6.7% respectively). Conversely, in Oro Province the rates were similar for male adults (aged 15-59) and youths (aged 15-24) (10% vs. 8.5% respectively). For females in the general population, the results are different. In Southern Highlands Province, similar proportions of female adults and youths reported a lifetime history of HAI (20.8% vs. 23.4% respectively). This contrasts with female youths and adults in East New Britain Province (13.9% vs. 6.2%) and in Simbu Province (15.4% vs. 5.7%). Condom use during lifetime HAI was not reported.

In the only study on the sexual practices of people with HIV, a minority (8.8% n = 11) reported engaging in HAI in the last six months [64]. Of these, only two people reported using a condom the last time that they had HAI with their regular partner. In contrast, 62.2% (n = 73) of participants reported condom use at last vaginal intercourse with their regular partner. It is unclear from the report if the sexual partner was also HIV-positive, therefore reducing the importance of condom use (and because all people in the study were on treatment). While the numbers on HAI in this study are small, it does suggest that people living with HIV may be a particular population warranting further education about the role of anal intercourse in the transmission of HIV (and other STIs).

### Risk equation analysis

**Figure 1:**
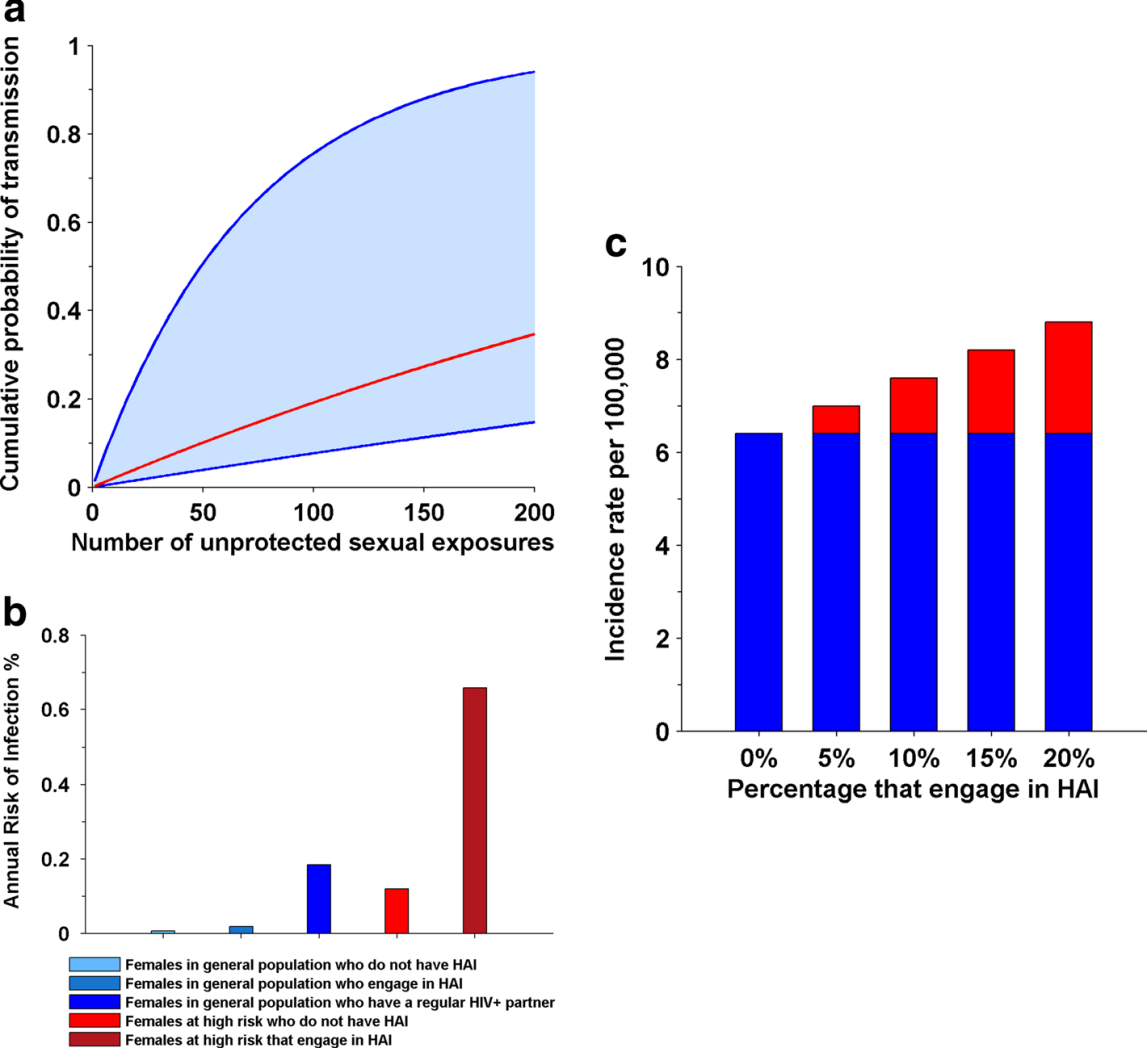
**Cumulative probability of HIV transmission over multiple sexual exposures for all females (a).** The lower line is the risk if all exposures involve vaginal intercourse. The upper line is the risk if all exposures involve HAI. The red line is the risk if 10% of exposures involve HAI. The risk females acquire HIV each year **(b)**. Females in general population with a HIV-positive regular partner assumed to have 100 sexual acts per year. HIV incidence rate in females in general population **(c)**. Red part of each bar represents the increase in incidence due to HAI.

HAI greatly increases the risk of HIV acquisition in females who practice it and could have a substantial impact on the HIV epidemic in PNG. Using the parameter values in Table 1, Figure 1a shows the cumulative probability of acquiring HIV through multiple unprotected exposures.

The risk equation analysis showed that the annual risk of acquiring HIV varies greatly across different female population groups (Figure 1b). In females from the general population who have HAI, if 10% of their sex acts involve anal intercourse, then their annual risk of acquiring HIV is 2.9 times higher than if they do not have HAI. For females at high risk, engaging in HAI increases the risk of HIV acquisition by 4.7 times. Females who have HAI and are in regular partnerships with HIV-positive males are at substantial risk of HIV having an annual risk of acquisition similar to FSWs who do not have HAI (Figure 1b).

Even if only 20% of females engage in HAI with only 1 in 10 of their sexual acts involving HAI, the total number of HIV infections in females will be 1.4 times greater than if only vaginal intercourse occurred (Figure 1c). Approximately 42% of new HIV infections in females will occur in the 20% of females who engage in HAI.

## Discussion

### Implications for surveillance and research

This systematic review helps to highlighting the complexity, diversity and heterogeneity of HAI in PNG according to both geographical location and by epidemiological sexual risk categories. Publication bias was unlikely to have been significant in this systematic review, as both published and unpublished data were located. However, key limitations of the research in PNG to date emerged from this systematic review. Although it is acknowledged that heterosexual transmission is the primary mode of HIV infection in PNG there is almost no understanding of the role that different sexual behaviours play. While there have been calls to conduct ‘further research into sexuality and sexual practices, and the meaning and ideologies behind these’ [67], the emphasis has been on concurrent and multiple sexual relationships and sexual networks rather than HAI [67]. Subsequently, few behavioural studies report explicitly on or discuss HAI generally and unprotected HAI specifically. Several studies used non-specific questions about sexual intercourse; for example, they would ask, ‘Have you ever had sex?’ or, ‘Last time you had sex did you use a condom?’ [68,69]. As a result, it can only be inferred which specific sexual behaviour is being described and measured.

Other included studies that addressed anal intercourse only asked if a person had ever had anal sex without specifying with whom one had anal sex with (i.e. for men was it with males, females or both males and females). With females this inference is less problematic (although not entirely). Indeed the standardised STI client record form used in government health facilities in PNG only asks men if they have had anal intercourse and does not specify with whom, and if with another male, it does not stipulate if it was receptive, insertive or both receptive and insertive. There were other studies however that conflated all anal sex together. For example, in one study the authors reported that 59% of male truck drivers had anal sex with a man or a woman in the past 12 months [62]. However, when read in conjunction with the complete findings, no male truck drivers reported having ever had sex with another male. Therefore, it appears that the anal sex reported is in fact HAI. Similarly, 31.4% of military men report anal intercourse of some kind but only 1% report having had sex with another male [62].

No study reported the frequency of HAI. HAI was rarely disaggregated by sex partner type (casual vs. regular). Studies seldom recorded condom use during HAI either by last HAI occasion or by type of sexual partner. We found no studies reporting the reasons for unprotected HAI. Another key limitation identified as a result of the systematic review is the discrepancy in recall periods used to measure HAI. Recall periods included lifetime, the last 12 months, the last 6 months, the last 3 months and the last month thereby making it difficult to compare data across studies and therefore conduct a meta-analysis.

Very little behavioural research has been conducted with people with HIV. Understanding the sexual practices of this population is critical to informing national HIV and STI prevention and treatment policy and programing, especially as treatment is being scaled up.

Few behavioural studies in PNG include qualitative data on HAI [42,64]. Therefore, the meaning and cultural context of these behaviours remain unclear, including what role (if any) sexual violence, traditional notions of pollution and conception and access to pornography (‘blue movies’) play in HAI. Nevertheless, the studies reviewed indicate men and women will talk about HAI when asked. Therefore, contrary to popular belief in PNG about people’s reluctance to talk about sensitive matters, when carefully and skilfully asked, people do talk about matters of sexual behaviour. This applies both to research but also clinical care.

To improve HAI reporting in PNG we recommend the following: ensure that questions are explicit about the type of sex partner and act carried out; standardised recall periods; and the documentation of condom use for HAI by partner type, last act and consistency of use (with the same recall period as for vaginal intercourse reporting). Furthermore, the number of sex acts per sexual behaviour in a given period is required. These data would help inform future public health campaigns to prevent HIV and other STI acquisition through unprotected HAI. In order to inform culturally appropriate condom use campaigns for HAI, reasons for not using condoms during HAI is also required.

### Implications for a policy and programmatic response

Until recently, HAI has been absent from key PNG policy documents. Fortunately, the most recent *National HIV and AIDS Strategy 2011-2015*[70] and the *National Gender Policy and Plan on HIV and AIDS 2006-2010*[71] acknowledge HAI. These documents recognise the importance of both heterosexual and homosexual anal intercourse and the training of health care workers to identify STI infection in women as a result of anal intercourse. However, this acknowledgment has not been translated into HIV and STI prevention efforts nor has it improved the detection, treatment and care of anorectal STIs. There is no data in PNG on anorectal STIs among women (or men who have sex with men). Although we argue that addressing structural drivers is essential to addressing HIV and STI risk and vulnerability, we believe that greater sexual health literacy is urgently needed in relation to HAI. This literacy is needed for key affected populations, the general population and health care workers providing sexual health services.

Although we assessed the risk that HAI poses for HIV transmission rather than for the transmission of other STIs, given that rates of STIs in PNG far outweigh HIV, we can safely assume that unprotected HAI also poses a large impact on STI incidence at the population level. While treating vaginal and penile STIs might be expected to cure most anorectal STIs (for instance, assuming the same pathogen is present at both sites), other infections may require site-specific diagnosis and treatment (for example, if several different pathogens are present or where an anorectal STI is present without a genital STI). We recommend that anorectal STIs be given research and clinical attention in PNG. Although STI clinics should routinely ask questions about anal intercourse in clinical assessments, this is not the practice in all clinics. An independent review by Save the Children in PNG’s *Poro Sapot Project* came to similar conclusions [72]. It highlighted that the national response inadequately addressed risk behaviours such as anal intercourse by both men and women and the lack of knowledge about the risks of HAI. They suggested that this may result in individuals viewing safe sex as unnecessary for anal intercourse even when they consistently use condoms for vaginal intercourse.

## Conclusions

In order to know your HIV epidemic, you should know the sexual behaviours and the socio-cultural meanings attached to them. This is particularly true in PNG where sexuality as well as sexual behaviour and practices are diverse and differ in many important ways from other countries. However, just as in many other parts of the world, HAI is commonly practiced in PNG. In PNG, HAI can have a large impact on HIV incidence amongst females from both key affected populations and the general population.

Studies have reported high rates of HAI across a diversity of populations, indicating that HAI is not limited to key affected populations. Despite the widespread practice of HAI in PNG, HAI has been a relatively neglected sexual behaviour and therefore mode of transmission for HIV and other STIs. The findings of this systematic review and mathematical modelling have a number of important implications. First, this review highlights the inadequacy of current knowledge of HAI and its role in the transmission of HIV and other STIs. Two, there is a need to re-appraise routine surveillance systems and behavioural research in order to ensure more robust and meaningful estimates of the prevalence of HAI including unprotected HAI and their contribution to HIV and other STIs. This would also entail enquiring about symptoms of anorectal STIs and not just genital symptoms of STIs. Three, increased efforts for the prevention, detection and treatment of anorectal STIs is needed, particularly among women involved in the selling and exchanging of sex and other women who report HAI. This would require health care workers to be skilled in enquiring about HAI and trained in how to identify and manage anorectal STIs in accordance with national STI treatment guidelines. Four, developing culturally appropriate and evidence-based HIV and STI prevention campaigns in PNG requires an understanding of the cultural meanings and context(s) of such practices. It is beyond the scope of this systematic analysis to offer insights into the meaning of HAI in PNG. As an outcome of this review a number of the authors (AK and AV) and other colleagues will undertake an inter-disciplinary study on anorectal STIs in PNG which will include, for the first time, the collection of anorectal specimens for the diagnosis of anorectral STIs in PNG. The results of this review, as well as planned research, will facilitate a more comprehensive understanding of HAI in order to improve the sexual health outcomes of people in PNG.

In conclusion, our systematic review shows that in PNG HAI is commonplace and condom use during HAI is universally low. Furthermore, results from our risk equation analysis show such practices could have a large impact on HIV incidence at the population level. Therefore, left unaddressed at all levels of the national response to HIV and STIs, unprotected heterosexual anal intercourse will continue to adversely affect the sexual health of Papua New Guineans, particularly women.

## Competing interests

The authors declare that they have no competing interests.

## Authors’ contributions

AKH conceived this paper, undertook the systematic review, drafted the manuscript and revisions and approved the final submission. RG conducted the risk equation, designed figures, significantly edited the manuscript, assisted in revisions and approved the final submission. WYNM and DW reviewed the risk equation, reviewed the manuscript and approved the final submission. AV and GL reviewed the manuscript and approved the final submission.

## Pre-publication history

The pre-publication history for this paper can be accessed here:

http://www.biomedcentral.com/1471-2458/13/1108/prepub

## Supplementary Material

Additional file 1 PRISMA flow diagram: Search strategy, inclusion and exclusion criteria and final results. Click here for file
