# Supplementary material for: A systematic review of heterosexual anal intercourse and its role in the transmission of HIV and other sexually transmitted infections in Papua New Guinea
Source: BMC Public Health. 2013 Dec 1;13:1108. doi: 10.1186/1471-2458-13-1108 (PMC4219522; doi:10.1186/1471-2458-13-1108)
Supplement: Additional file 1 — PRISMA flow diagram: Search strategy, inclusion and exclusion criteria and final results. [file 1471-2458-13-1108-S1.pdf]

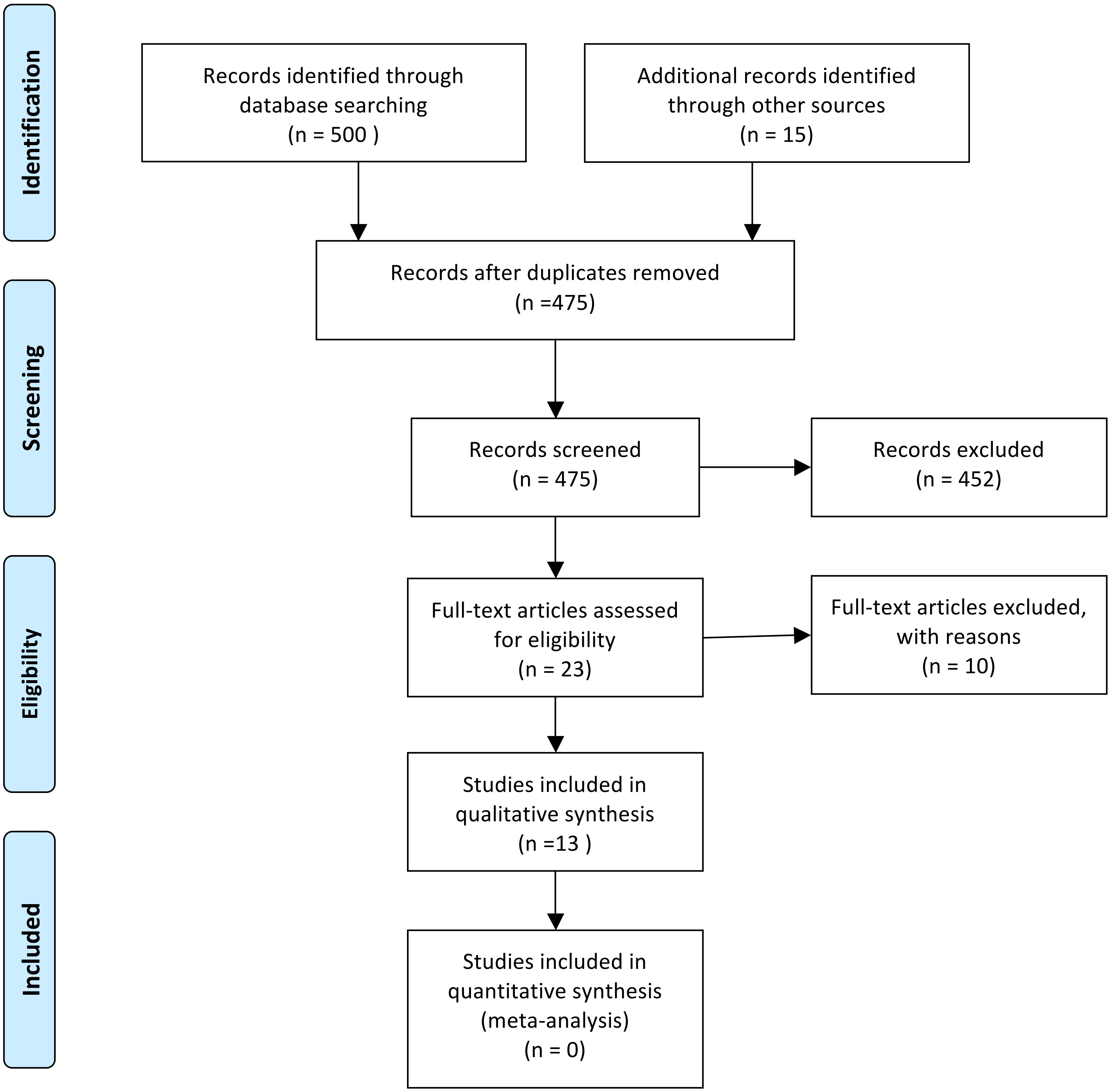
Identification
Screening
Eligibility
Included
Records identified through database searching (n = 500 )
Additional records identified through other sources (n = 15)
Records after duplicates removed (n =475)
Records screened (n = 475)
Records excluded (n = 452)
Full-text articles assessed for eligibility (n = 23)
Full-text articles excluded, with reasons (n = 10)
Studies included in qualitative synthesis (n =13 )
Studies included in quantitative synthesis (meta-analysis) (n = 0)
